# National multiple myeloma cohort: Gaps and opportunities for research in Brazil

**DOI:** 10.1016/j.htct.2025.106229

**Published:** 2025-12-12

**Authors:** Diogo Moreira do Amaral

**Affiliations:** Universidade São Francisco (USF), Bragança Paulista, SP, Brazil

Dear Editors,

Multiple myeloma (MM) is a relatively uncommon hematologic malignancy. It accounts for approximately 1 % of all cancers and is the second most frequent hematologic malignancy [1]. However, national data on the epidemiological profile, access to innovative therapies, and clinical outcomes in Brazil remain scarce [2].

The study “Overall survival in multiple myeloma in Brazil: A cohort of 16 years”, published in Hematology, Transfusion and Cell Therapy, provides a comprehensive overview of survival among MM patients in the country. This study evaluated 25,370 patients treated within the Brazilian Unified Health System (SUS) between 2000 and 2015, constituting one of the largest national MM cohorts ever published. It allows for important reflections on gaps and opportunities in research and health policy [3].

The epidemiological data of the study align with the national literature. Key characteristics included: a median age of patients of 62 years, a predominance of males, regional differences (highest concentration in the southeastern region), and overall survival (OS) of 37 months. These findings are consistent with other Brazilian analyses [4]. but contrast with countries such as the USA and France, where the median age at diagnosis is higher (66–74 years) and survival is longer [5,6]. This underscores differences in demographics, epidemiology, and access to therapy.

The study also evaluated the impact of different therapeutic regimens. Although bortezomib (Bortezomib) was only formally incorporated into the SUS in 2020, patients treated with this agent had an OS of 67 months, while those on thalidomide-based regimens reached 54 months. Notably, patients undergoing hematopoietic stem cell transplantation (HSCT) exhibited an even higher survival, with a median of 87 months [3]. These results reinforce the clinical benefits of innovative therapies and transplantation, highlighting the importance of early access to effective treatments.

However, despite the robustness of the administrative database used by the authors (DATASUS), some gaps remain. There are no data on treatment adherence, toxicity, cytogenetic stratification, or responses to specific lines of therapy. Additionally, the absence of information on time to diagnosis, functional status, and associated comorbidities limits individualized interpretation and prevents understanding the determinants of survival. Furthermore, the methodology adopted—classifying patients according to therapy exposure at any point rather than only the first line regimen—reflects real-world complexity, although it may introduce selection bias.

The difference in survival of individuals undergoing HSCT underscores its relevance and efficacy as a standard of care. However, it is noteworthy that HSCT was performed in only 26.9 % of the population, despite its established efficacy as a standard therapeutic cornerstone for eligible patients [3]. In the Brazilian context, this low rate reflects structural barriers, including an insufficient number of specialized centers, regional disparities in healthcare access, long waiting times, and socioeconomic limitations. Thus, concerns remain regarding the underutilization of transplantation in Brazil [7]. Additionally, the late incorporation of new drugs, such as bortezomib (Bortezomib), demonstrates the need for more agile health technology assessment processes without compromising safety and efficacy [8]. Consequently, it is plausible that many eligible patients never undergo HSCT, limiting the potential gains shown by the national cohort.

Importantly, this study offers key lessons for Brazil. In summary, three points should be considered: there is room for more detailed investigations that can translate evidence into concrete improvements in clinical practice; it is essential to discuss strategies that expand equitable access to HSCT, such as increasing the network of specialized centers, coupled with rapid incorporation policies and adequate funding. Robust national data can support health policy, guide therapeutic protocols, and reduce reliance on evidence from other countries, which often have distinct population profiles.

In conclusion, the 16-year national cohort analysis highlights the impact of innovative drugs, the importance of long-term studies for MM in Brazil, exposes failures in access to innovative therapies and HSCT, and underscores the urgency of public policies capable of democratizing this access. Encouraging the collection of population-based data, expanding access to medications, and increasing the transplantation network are essential steps to improve care for patients with MM in Brazil.

## Data availability statement

The data that support the findings of this study are available from the corresponding author upon reasonable request.

## Conflicts of interest

The authors declare no competing financial interests.
